# Biophilia as Evolutionary Adaptation: An Onto- and Phylogenetic Framework for Biophilic Design

**DOI:** 10.3389/fpsyg.2021.700709

**Published:** 2021-07-21

**Authors:** Giuseppe Barbiero, Rita Berto

**Affiliations:** GREEN LEAF - Groupe de Recherche en Education à l’Environnement et à la Nature, Laboratorio di Ecologia Affettiva, Università della Valle d’Aosta - Université de la Vallée d’Aoste, Aosta, Italy

**Keywords:** biophilia hypothesis, biophilia ontogenesis, biophilia phylogenesis, biophilic design, biophilic temperament

## Abstract

Biophilia is a human personality trait described initially by Erich Fromm and later by E.O. Wilson, both of whom agree that biophilia has a biological basis and that it is fundamental to develop harmonious relationships between humans and the biosphere. This review aims at establishing a definition of biophilia as an evolutionary process. To this end, the most significant studies of evolutionary psychology were considered, to outline the fundamental characteristics of a hypothetical biophilic temperament/personality and to reconstruct a plausible history of biophilia as an evolutionary process. This process considers different typologies of Nature (wilderness, rural, and urban) and human cultures (Paleolithic, Neolithic, and Burg) and leads us to consider environmental preference and psycho-physiological recovery in relation to the threshold of time spent in contact with Nature. Unfortunately, modern people, especially children, lack direct and frequent contact with Nature and this can have negative consequences on their physical and mental health. Biophilic design, considering the evolutionary roots of this architectural approach, is an effective way of planning/designing interior and urban environments to stimulate the innate biophilia of the individual.

## Introduction

Biophilia is a combination of two words that descend from ancient Greek: “life” (bio) and “love” (philia); it literally means love of life. When we talk about “love of life,” it is appropriate first of all to specify what we mean by “life.” In a recent article, Lenton et al. (2020) proposed to indicate with the word *life* (spelled out all in the lower case) the class of properties that are common to all living beings, and with the word *Life* (capitalized) the phenomena that emerge from the coupling of the metabolism of living organisms with the external environment. In this sense, *Life* appears as a dynamic process in which organic and inorganic forces concur in continuously remodeling Gaia’s habitable conditions (Volk, 1998; Lenton and Watson, 2011); according to Gaia hypothesis, Earth is a self-regulating system in which the biota play an integral role (Kump et al., 2010). Human beings are capable of recognizing living organisms (*life*) in the non-living world as well as Nature (*Life*) as a process in its entirety (Barbiero, 2014). Therefore, biophilia can mean both love for living creatures (*life*) and love for Nature (*Life*), understood as the set of living creatures *plus* the abiotic environment in which they thrive. In this paper, “Nature” is written with the capital “N” to indicate the biosphere and the abiotic matrices (soil, air, and water) where it flourishes, and to avoid confusion with “nature” as the intrinsic quality of a certain creature and/or phenomenon.

The word “biophilia” was coined twice independently by German psychologist Erich Fromm and American biologist E.O. Wilson. Fromm used the term biophilia to describe the *psychological orientation* of being attracted to all that is alive and vital (Fromm, 1964), thus assuming a predominantly *ontogenetic* perspective, aimed at understanding the conditions for developing a biophilic personality. Wilson used the term biophilia to describe the traits of *evolutionary adaptation* that allow us to develop a mental link with the living world and Nature (Wilson, 1984), thus assuming a predominantly *phylogenetic* perspective. Later on, Wilson, together with Stephen R. Kellert, published the collection of essays *The Biophilia Hypothesis* (Kellert and Wilson, 1993); this hypothesis asserts the human dependence on Nature “that extends far beyond the simple issues of material and physical sustenance to encompass as well the human craving for aesthetic, intellectual, cognitive, and even spiritual meaning and satisfaction” (Kellert, 1993, p. 21). The biophilia hypothesis must be compatible with our knowledge of evolutionary biology and psychology to make it possible to reconstruct a plausible and coherent history of biophilia with what we know of Nature in the Pleistocene and Holocene eras, and of our cultural evolution in the Paleolithic (Tattersal, 2008) and most importantly in the Neolithic era (Diamond and Bellwood, 2003), when our relationship with Nature changed radically.

In this review, we aim to establish a definition of biophilia as an evolutionary phenomenon, while highlighting three areas that need further research: the typology of the natural environment, the typology of human experience, and the exposure threshold (1) *The typology of the natural environment.* Nature is not the same everywhere; classification criteria of the different natural environments are necessary for them to be related to environmental preference and any psycho-physiological effects (2) *The typology of human experience with the natural environment*. Our species has developed a series of adaptations to the *quality* and *quantity* of Nature which are part of our evolutionary history. Our ancestors survived in the Pleistocene wilderness, they learnt to domesticate animals and plants in the Neolithic, and today, they live in urban environments where Nature is almost disappeared. Recognizing these adaptations throughout our evolutionary history as a species could help us understand the development of the broad spectrum of psycho-physiological reactions that Nature elicits in us (Wilson, 1993) (3) *The threshold of exposure to the natural environment*. How much Nature is needed and for how long to obtain subjective and/or objective benefits? This question takes on dramatic importance in an era where urbanization has deprived entire generations of direct contact with Nature, leading to problematic phenomena of disconnection from Nature (Chawla, 2016). Besides the various suggestions on how to alleviate or reverse this trend, it is also essential to learn how to design urban spaces where contact with Nature is continuous and sustained. As we will see in Section “Biophilic Design For a Biophilic Environment,” the goal of *biophilic design* is to give back to human beings living in urban environments the possibility of maintaining contact with Nature (Kellert, 2008).

## The Interpretations of Biophilia

### Erich Fromm’s Ontogenetic Perspective

Erich Fromm was the first to introduce the term *biophilia*, defined as “the passionate love of life and of all that is alive” (Fromm, 1973, p. 406). According to Fromm, biophilia manifests as the “wish to further growth, whether in a person, a plant, an idea, or a social group” (Fromm, 1973, p. 406) and includes “love for humanity and nature” (Fromm, 1994, p. 101). The concept of biophilia was developed at various times by Fromm (1964, 1973), to describe the mental tendency to preserve life and fight death (Fromm, 1964). The fundamental trait of biophilia is “the experience of union with another person, with all men, and with nature, under the condition of retaining one’s sense of integrity and independence” (Fromm, 1955, p. 37). Biophilia restores “an active power in man” which “makes him overcome the sense of isolation and separateness” (Fromm, 1956, p. 17).

Fromm recognizes the innate and ontogenetic character of biophilia, as a “primary potentiality” (Fromm, 1964, p. 51) “intrinsic to human biology” (Fromm, 1973, p. 407). However, Fromm warns that biophilia is realized only if the environmental conditions – natural and social – are present that foster its growth and development in a natural and spontaneous way. If the environmental conditions are in contrast with existential needs, then an opposite *secondary potentiality* may develop, necrophilia, that is, “the passion to destroy life and the attraction for all that is dead, in decay and purely mechanical” (Fromm, 1973, p. 25). According to Fromm, “there are three environmental conditions that must precede the development of biophilia: security, justice and freedom” (Fromm, 1964, p. 52). To develop a biophilic orientation, the first environmental condition is physical and mental *security*. These require that there be shelter as well as access to economic resources. Conversely, insecurity and economic scarcity encourage necrophilia. An individual or group forced to “ward off starvation” will not develop a biophilic orientation (Gunderson, 2014). The second environmental condition is *justice*. Living in a context in which behavioral rules are observed, including duties and expectations for oneself and others, fosters a biophilic attitude. Biophilia flourishes in a context where an individual can decide to think, express him of herself, and act without constraints, being able to devise and implement actions with *free* choice of ends and tools that he or she deems useful to achieve his or her goals. If the preconditions for biophilia are met, then it is possible to cultivate four active elements for biophilia: care, responsibility, respect, and knowledge (Fromm, 1956). Unfortunately, Fromm developed the description of the active elements for biophilia only as far as the relationships between human beings, with the only exception of the *care* element, which Fromm extended also to Nature. Ryan Gunderson (2014) has proposed a plausible extension of Fromm’s concept of biophilia in relation to Nature. According to Gunderson, Fromm’s love of Nature means (1) an active concern for the growth and prosperity of Nature, (2) the ability to respond to and satisfy the needs of Nature, (3) respect for the autonomy and independence of Nature and its defense from human interests, and (4) having knowledge of Nature without dominating it.

### E.O. Wilson’s Phylogenetic Perspective

E.O. Wilson’s (1984) biophilia hypothesis adopts an evolutionary interpretation and offers a phylogenetic perspective to our love for life. Wilson defines biophilia as “our innate tendency to focus upon life and life-like forms and, in some instances, to affiliate with them emotionally” (Wilson, 2002, p. 134). In this form, it is already an operational definition, because it identifies two fundamental constructs of biophilia: fascination (*focus upon*) and affiliation.

Nature exerts a *fascination* on human beings, that is, an attraction capable of activating the involuntary/effortless modality of attention. Fascination is the key concept of the Attention Restoration Theory (ART; for more details see Kaplan, 1995). People respond with involuntary attention to natural settings, and this guarantees that directed attention can rest and be restored from mental fatigue in adults (Berto, 2005) and in children (Barbiero et al., 2014). Nature represents a fascinating stimulus of choice (Kaplan, 1995, 2001).

*Affiliation* for Nature is an emotional bond with specific forms of life that takes place in certain circumstances (Wilson, 2002, p. 134). From an evolutionary point of view, the feeling of affiliation seems to reside in “our capacity to experience empathy with other creatures and respond to their concerns as our own” (Goodenough, 1998, p. 127). Empathy, which can be a mediator of affiliation with Nature (Di Fabio and Kenny, 2018), is “an emotional state triggered by another’s emotional state or situation, in which one feels what the other feels or would normally be expected to feel in his situation” (Hoffman, 2008). Normally, a feeling of empathy develops between two human beings; however, the ability to experience empathy is not limited to humans (Angantyr et al., 2011). Forms of differentiated emotional participation and affective empathy are widespread in mammals (Preston and de Waal, 2002). Domestic Nature offers ample possibilities for empathic contact (Hand et al., 2017a) and can help reduce the stress response, as suggested by the Stress Recovery Theory (SRT; for more details see Ulrich et al., 1991). It is therefore reasonable to assume that more frequent contact with domestic Nature tends to aid a faster recovery from stress (Ulrich, 1984). The main empirical evidence of the relationship between the feeling of affiliation and recovery from stress comes from studies on relationships with pets (Coakley and Mahoney, 2009). Humans like to establish emotional relationships with their pets, because this type of affiliation reduces the stress response (Sapolsky, 2004, pp. 234–248; Kertes et al., 2017).

According to Wilson, “biophilia is not a single instinct but a complex of learning rules that can be teased apart and analyzed individually. The feelings molded by the learning rules fall along several emotional spectra: from attraction to aversion, from awe to indifference, from peacefulness to fear-driven anxiety” (Wilson, 1993, p. 31). Two considerations follow from this: (1) biophobia – meaning “fear of or strong negative/avoidance responses to certain natural stimuli that presumably have constituted risks during evolution” (Ulrich, 1993, p. 76) – is an intrinsic and complementary part of biophilia and (2) biophilia is innate but not instinctive (Lee, 2012). It is innate insofar as it is a manifestation of a phenotype that has passed the scrutiny of natural selection and can be studied from a phylogenetic perspective. It is not instinctive because it does not give rise to rigid and deterministically fixed behavior (Wilson, 1993). Over the course of evolution, biophilia has become part of the human genotype (Kellert, 2009), through a process of co-evolution of culture and genes (Wilson, 1993) which has bestowed an advantage in terms of real fitness to individuals capable of becoming emotionally affiliated with the environment (Kellert, 1997).

### Biophilia: State or Trait?

Fromm and Wilson offer two complementary perspectives of biophilia, which together define a theoretical horizon for the experimental verification of the biophilic hypothesis. In many cases, the two perspectives mirror each other. For example, a biophilic personality has many good reasons to appreciate Nature. At any rate, Nature is more likely to fascinate a biophilic personality rather than a necrophilic/biophobic one. Fromm and Wilson agree that biophilia has a biological basis (Fromm, 1973; Wilson, 1993) and that it is a fundamental human force for developing harmonious relationships between humanity and the biosphere (Fromm, 1963, 1966; Wilson, 1984, 1993). However, there are substantial differences. Where Wilson defines biophilia in its biological terms which relate to humanity as a species, Fromm is more attentive to the environmental and social conditions that affect the individual developing biophilia. Wilson’s definition has the advantage of being more operational because it has identified the constructs of fascination and affiliation which have allowed the operationalization of biophilia on an evolutionary basis.

The phylogenetic interpretation allows us to understand how biophilia can become a “total orientation, an entire way of being” (Fromm, 1964, p. 45) which permeates the entire personality. Personality is defined as the set of inherited and acquired mental qualities that define an individual’s temperament and character through a process of adaptation as a compromise between internal needs and external demands (Cloninger et al., 1993). Many clues suggest that biophilia is a hereditary trait. Firstly, biophilia is considered intrinsic to human nature (Fromm, 1973; Wilson, 1984; Kellert, 1993; Gardner, 1999); accordingly, it can assume that biophilia is ubiquitous in human cultures and probably an “absolute universal,” a psychobiological trait that has been forged by evolution (Brown, 2004). Secondly, biophilia possesses the four characteristics considered typical of a temperament trait: It (1) is present since early childhood, (2) has its counterpart in animals, especially as a guide in finding shelters and resources, (3) is determined by innate biological mechanisms, and (4) is subject to changes caused by maturation and individual-specific genotype-environment interaction (Strelau, 1998). Therefore, biophilia could be a basic, relatively stable personality trait which expresses itself in one’s reactions and behavior when in contact with Nature.

Biophilia, however, consists of “weak learning rules” (Wilson, 1993, p. 32), which leave ample freedom to the individual. Every individual conduct has elements attributable to an innate heritage and a learned heritage and what is phylogenetically selected is a greater receptivity toward certain ontogenetic contingencies (Caprara and Gennaro, 1994). Plausibly, sequences of responses or behaviors are not what is inherited but rather a greater susceptibility to certain environmental stresses and to the possibility of establishing certain links between individual reactions and situational contingencies. Human behavior is not affected by instinct like that of animals. And here is where character comes into play, and all the environmental conditions influence it, mainly education. Fromm was clear on the function of character to “substitute for” the instinctive equipment that human beings lack (Fromm, 1973, p. 255). The innate component of biophilic behavior is resolved in the environment with which the genetic heritage interacts and from which it takes the forms to be translated into behaviors, dispositions, and personalities. Education is fundamental in the formation of character (Williams, 2000), and it may or may not help in the formation of a biophilic personality. It is clear, however, that education cannot go along without contact with Nature (Kahn, 2002), because biophilia is innate: “when human beings remove themselves from the natural environment, the biophilic learning rules are not replaced with versions equally well adapted to artifacts” (Wilson, 1993, pp. 31–32). Education could enhance the evolutionary core of biophilia, which consists of a set of *learning rules* which facilitate a faster and more effective ability to interact with the environment. Biophilia could be defined as an innate predisposition to learn from and interact with Nature. Learning predispositions are of great importance for the *Homo sapiens* species. Children are extraordinarily inept at birth and go through a very long phase of learning and inculturation, during which they learn the correct behaviors (Tizard and Hughes, 2008), including those relating to Nature (Klaar and Öhman, 2014). A fast and effective learner has an evolutionary advantage, which continues to be appreciated in all school systems (OECD, 2015). Therefore, evolution may have fostered not only innate biological mechanisms to be able to relate to Nature, but also the quick learning of its “laws” (Meyer, 1997; White and Stoecklin, 1998).

## Biophilia as an Evolutionary Adaptation

Over the course of evolution, humanity has had to face many hostile forces of Nature and it is reasonable to think that the “natural selection should have favored individuals who were motivated to explore and settle in environments likely to afford the necessities of life but to avoid environments with poorer resources or posing higher risks” (Orians and Heerwagen, 1992, p. 557). The phylogenetic approach suggests that the learning rules of biophilia may have become rooted in the gene pool of our species in relation to the contribution which they have given and can still give to improving human adaptation to the environment.

### Phylogeny of Biophilia

The biological evolution of our species took place in the wilderness, the Nature of the Late Pleistocene. For about 95% of our evolutionary history, corresponding to the Middle Paleolithic and Upper Paleolithic Eras, humans have survived as hunters-gatherers. Humans have thus perfected a set of adaptive responses to different wild environments – mainly the savannah (Orians and Heerwagen, 1992) – aimed at recognizing the quality of an environment in terms of shelters and resources (Buss, 2016). Safe and resource-rich environments are a precondition for biophilia (Fromm, 1964); they reduce the stress response and promote the restoration of cognitive processes (for a review, see Berto, 2014). Some environmental preferences (Balling and Falk, 1982; Robinson and Breed, 2020) could therefore be the result of adaptations that proved effective in our ancestors’ struggle for survival (Falk and Balling, 2010). Furthermore, recovering from mental fatigue in a shorter time may have provided an additional evolutionary advantage (Kaplan and Kaplan, 1989).

The relationship with Nature changed in the Neolithic, which covers approximately 5% of humanity’s evolutionary history (Larson et al., 2014; Stephens et al., 2019). After the invention of agriculture (Purugganan and Fuller, 2009) and animal breeding (Larson and Fuller, 2014), about 14,000 years ago (Arranz-Otaegui et al., 2018), humans began to distinguish domestic Nature (good) from wild Nature (bad). The biophilic trait may have entered an adaptation and exaptation cycle (Gould and Vrba, 1982) to develop new forms of adaptation and promote its better use based on the demands of the new Neolithic lifestyle.

With the Industrial Revolution, starting from the second half of the eighteenth century – an irrelevant period from the point of view of evolutionism, corresponding to less than 0.1% of the history of humanity – humans began to create urban environments, characterized by an increase in population density and a decrease in green spaces (Szreter and Mooney, 1998). During this period, the size of urban agglomerations grew, and now, they are inhabited by more than half of the world’s population (Worldbank, 2019). In urban environments, visible Nature has almost disappeared, even if not completely (Beatley, 2011) and consequently, the natural stimuli useful for developing biophilia have been reduced (Berto and Barbiero, 2017a).

The Neolithic age and especially the Industrial Revolution, which fueled the urbanization process, led to two moments of rupture with Nature: first with wilderness and then again with domesticated Nature. Although these two moments of rupture have strongly influenced the processes of inculturation, the predisposition to learn from Nature has probably remained the same (Wilson, 1993). What has changed is the nature of the Nature to learn from; however, many evidences indicate that the imprint of wilderness has remained deep within the human psyche (Estés, 1992).

### The Typology of the Natural Environment

Nature is not the same everywhere. Some types of Nature seem to stimulate biophilia and are preferred, while other types of Nature seem to stimulate biophobia and are avoided (for more details, see Kaplan and Kaplan, 1989). In general, people seem to become more fascinated by the kind of Nature that matches their feeling of affiliation (Fredman and Emmelin, 2001; Van den Berg and Koole, 2006; Nisbet and Zelenski, 2011); thus, people who have a high affiliation with Nature prefer wilder natural environments (Berto et al., 2018; Løvoll et al., 2020), while people with a lower affiliation tend to prefer more domestic natural environments (Bixler and Floyd, 1997; Davis and Gatersleben, 2013).

Since biophilia appears primarily as a functional evolutionary adaptation to an environment characterized by wild Nature, any criteria for classifying natural environments must start from a precise definition of wild environment, that is, “an area where the earth and its community of life are untrammeled by man, where man himself is a visitor who does not remain” (Wilderness Act, 1964). Berto et al. (2018) is one of the few studies which used a criterion for evaluating the wildness level of a natural environment, specifically, the Recreation Opportunity Spectrum (ROS, Clark and Stankey, 1979) adapted to the European Union context (Paracchini et al., 2014). ROS is a management tool which classifies natural areas according to their recreational opportunities. To evaluate wilderness levels, ROS uses two general criteria: the *accessibility* and *social experience*, which can be had in an environment. Accessibility depends on the *extent* of the area considered, the type of *access*, *naturalness*, based on preservation criteria that evaluate how much the environment has been modified by human hands, and *remoteness*, that is, the distance from access routes. The social experience depends on the number of *social encounters*, available *facilities*, *visitor impacts*, and the *visitors themselves*. Based on these parameters, ROS identifies seven environments in service use: urban (U), rural (R), roaded modified (RM) in some areas, roaded natural (RN), semi-primitive motorized (SPM), semi-primitive non-motorized (SPNM), and primitive (P). A natural environment is defined as “wilderness” when it extends over more than 20 km^2^, the nearest road is at least 5 km away, and one can meet less than six people per day or less than three visitors at a campsite. Using ROS to distinguish the degree of naturalness of four parks, Berto et al. (2018) found that park visitors with a higher affiliation with Nature (measured by the Connectedness to Nature Scale; Mayer and Mc Pherson Frantz, 2004) had a perception of greater restorative benefits (measured by the Perceived Restorativeness Scale; Hartig et al., 1997) in parks with a higher degree of naturalness. ROS is built on an empirical basis; however, in defining a gradient of naturalness, it follows in detail the model “Paleolithic (Wild Nature: P, SPNM) – Neolithic (Domestic Nature: SPM, RN, RM, and R) – Urban (Nature absent in the Burg: U),” which is useful in offering a classification of the environments, having allowed us to predict the effects of the environment type based on the individual link with Nature (Table 1).

**Table 1:** Individual’s preference for Nature typologies (the first two columns from the left) affects the individual’s perception of the benefits to obtain in terms of subjective wellbeing (the last column on the right), and this perception is mediated by the individual’s connection to Nature (the third column from the left).

Nature typologies	ROS classification	Affiliation with nature	Perceived restoration
Wilderness (Paleolithic)	P, SPNM	High	High
Rural (Neolithic)	SPM, RN, RM, R	Medium	Medium
Burg (Urban)	U	Low	Low

*U, urban; R, rural; RM, road modified; RN, roaded natural; SPM, semi-primitive motorized; SPNM, semi-primitive non-motorized; and P, primitive*

Although the existing literature invites us not to underestimate the importance and value of domestic Nature, it is evident that the landscapes and green spaces with low population density and greater naturalness have a high restorative power which is immediately perceived (Kuo, 2015), and thus gain one more reason to be preserved in their naturalness.

### The Typology of Human Experience With the Natural Environment

Why does a desire for wild Nature correspond to a higher affiliation with Nature (see Table 1)? The answer may once again be evolution. Biophilia presumably evolved and was successful in the Pleistocene, when *only* wild Nature existed. However, the human evolutionary experience with Nature had at least two important moments of rupture: the Paleolithic-Neolithic transition (Ellis et al., 2016) and the Neolithic-Urban transition (Schultz, 2002). A successful adaptation as far as the relationship with Nature in the Paleolithic may no longer be as effective in the Neolithic, when domestic Nature is the prevailing environment with which humans interact. Some manifestations of biophilia likely continued to be suitable both for Paleolithic hunters and Neolithic breeders, while other manifestations of biophilia may have been suitable for both Paleolithic gatherers and Neolithic farmers. However, when humans learned to cultivate plants and breed animals, that is, to transform a part of wild Nature into domestic Nature, it is likely that Wild Nature was perceived as an “enemy,” to be pushed away and rejected, and feelings of affiliation would then be reserved only for domesticated plants and animals (Cronon, 1996). One example is our relationship with the *Canis lupus* species. The wolf is the wild version of *C. lupus*, and it was the only form known to Paleolithic humans, who feared and admired the wolf, so much so that they made it into an archetype (Jürgens and Hackett, 2017). Neolithic humans continued to fear and admire the wolf, but they rejected it, while protecting the dog, the domestic variant of *C. lupus*, because it was useful to the new lifestyle.

Biophilic traits could have pleiotropic characteristics; in practice, the same adaptation could prove useful in a different context thanks to an exaptation cycle (Gould and Vrba, 1982). Affiliation with wild Nature could be a “personality trait” with a pleiotropic effect on the perception of a restorative environment and the population density of a certain area. For a Paleolithic human, the usual landscape was devoid of visible places of human aggregation. It is therefore presumable that the restorative environment was perceived *without* such places. Opposite to that, the landscape of a Neolithic human was characterized by visible places of human aggregation, which were landmarks for orientation and often were the final goal of a transfer. It is therefore presumable that the restorative environment was perceived *with* such places.

However, an exaptation cycle does not always lead to an optimal result. Paleolithic humans lived in small nomadic communities roaming over large areas; population density was low and encounters outside one’s own clan were rare. On the other hand, Neolithic humans lived in stable villages and smaller areas, where population density was higher and encounters more frequent (Stiner, 2001). Village life requires a higher level of socialization and hitherto unknown physical proximity that turns out to be stressful (Larsen et al., 2019) and to which we still do not seem fully adapted. This could explain, for example, the stress response to crowding and why many people seek outdoor spaces in Nature where human presence is rare.

The pleiotropic effect appears more evident in the transition from the Neolithic to the Urban, that is, in the passage from the rural to the burg environment. In an Urban environment, the usual landscape is apparently devoid of Nature. Nature is almost invisible and cannot support restoration needs which are replaced by artificial surrogates (Galtung, 1984). The lifestyle changes and biophilia atrophies, but is never completely extinguished (Wilson, 1993). If this hypothesis is correct, then in each human being three fundamental experiences (and cultures) are probably present and settled: the wild Nature of the Paleolithic, the domestic Nature of the Neolithic, and the absent Nature of the Urban environment. Together, all three phylogenetic experiences of affiliation, with different intensities varying from individual to individual, give rise to a specific relationship with Nature (Barbiero, 2021).

### The Threshold of Exposure to the Natural Environment

The desire to establish and maintain contact with Nature probably depends on experiences of stress recovery (Ulrich et al., 1991; Berto, 2014; Lee et al., 2015; Martyn and Brymer, 2016) or attention restoration (Kaplan, 1995), or both (Cimprich and Ronis, 2003). This could explain why, in social relational contexts, environments in which Nature is present are preferred to environments in which it is absent (Lindemann-Matthies et al., 2010; Lin et al., 2014), especially when people engage in fun activities (Cleary et al., 2017). Natural environments are preferred for activities that aim to provide relaxation from daily routines, such as vacations and receptions, and often serve as the backdrop for social media sharing (Chang et al., 2020). It does not come as a surprise that humans tend to associate Nature with emotional happiness (White et al., 2013; Capaldi et al., 2014; Zelenski and Nisbet, 2014; Kuo, 2015; Biedenweg et al., 2017). Nature offers places where people can feel relaxed, forget their worries, and reflect on personal matters (Ouellette et al., 2005; Moreton et al., 2019a; Graves et al., 2020). Nature seems to satisfy the psychological need for belonging and relating (Moreton et al., 2019b); actually, activities that involved contact, emotional attachment, meaning, beauty, and a compassionate relationship with Nature are pathways for improving Nature connectedness, pro-environmental behavior, and wellbeing (Lumber et al., 2017). Studies show that in natural environments people tend to behave more altruistically (Weinstein et al., 2009; Zelenski et al., 2015; Guéguen and Stefan, 2016) and there is an increased sense of satisfaction with life (Biedenweg et al., 2017).

The beneficial effects of being in contact with Nature also depend on the time of exposure and the frequency of such contact. Several empirical observations show that the variables “time spent” and “frequency of contacts” affect the feeling of affiliation with Nature (Nisbet et al., 2009; Prévot et al., 2018; Bonnell et al., 2019). Experimental designs to verify the effect of “time” in Nature might be difficult to conceptualize and compare; they differ on the type of Nature contact (slide, video, walk, exercises, wilderness program, etc.) and on the psychological outcomes (questionnaire, self-rating scales, objective test, etc.); however, they suggest that individual’s response to Nature seems to be dose-dependent (for more details, see Kaplan and Berman, 2010). In fact, “negative” studies on the effects of Nature – that is, observing what happens when there is not direct and frequent contact with Nature (Schultz, 2002; Kesebir and Kesebir, 2017) – show that disconnecting from Nature has detrimental effects on both mental and physical health (Ulrich, 1993; Frumkin, 2001; Schultz et al., 2004). Many studies observe that direct exposure to Nature, even when brief (15 min), can offer visible psychophysiological benefits (Mayer et al., 2009; Mackay and Neill, 2010; Ryan et al., 2010; Nisbet and Zelenski, 2011).

A large quantitative dose-response study was recently carried out where the self-reported minutes spent in natural environments for recreation in the last 7 days were compared in relation to self-reported health and subjective wellbeing (White et al., 2019). This research revealed that there is a minimum “threshold” of exposure to Nature which can be quantified as 2 h per week. People who reported spending at least 2 h in Nature per week exhibited consistently higher levels of health and wellbeing than those who reported no exposure. It does not matter in which way the 2-h threshold was reached, whether by long weekend walks in places far from home or short and regular walks in urban parks, nor which activity took place in these 2 h immersed in Nature. Despite the numerous limitations recognized by the authors themselves, this research establishes a clear reference point for subsequent research, in relation to the dose of Nature as a function of people’s responses insofar as their perceived wellbeing.

## Building Environments That Stimulate Biophilia

### Nurturing Children’s Affiliation With Nature

The feeling of affiliation with Nature depends on how one sees, treats, and cares for Nature, animals, plants, and natural resources. It also depends on how familiar one is with natural environments, and the degree of comfort and wellbeing that one experiences in such environments (Capaldi et al., 2015; Korpela et al., 2018; Bratman et al., 2019). Although it is a rather complex biophilic construct, affiliation with Nature can be represented in operational terms and as such it can be measured by means of different approaches. At least 10 approaches have been suggested to describe and measure affiliation with Nature: Emotional Affinity Toward Nature (Kals et al., 1999); Inclusion of Nature in Self (Schultz, 2001; Martin and Czellar, 2016); Environment Identity (Stets and Biga, 2003); Environmental Identity (Clayton, 2003); Connectedness to Nature (Mayer and Mc Pherson Frantz, 2004); Self-Nature IAT (Schultz et al., 2004); Connectivity with Nature (Dutcher et al., 2007); Commitment to Nature (Davis et al., 2009); Nature Relatedness (Nisbet et al., 2009); and Love and Care for Nature (Perkins, 2010). Each of these approaches captures slightly different aspects of affiliation with Nature. At any rate, for all practical purposes, at least seven of these 10 modalities are quite equivalent (for more details see Tam, 2013).

The sense of affiliation with Nature matures quite early in childhood, following a rather precise value pattern (Kahn and Kellert, 2002) and development of the environmental cognition (Barbiero and Berto, 2016). The relationship between children and Nature has been extensively studied (Kahn, 1997; Kahn and Kellert, 2002; Gill, 2014; Adams and Savahl, 2017; Tillmann et al., 2018), and the consensus is almost unanimous that children’s first experiences with Nature are fundamental (Wells and Lekies, 2006; Dadvand et al., 2015), lacking which incompetence prevails (Balmford et al., 2002) together with a feeling of fear for Nature (Bixler and Floyd, 1997). Children generally appreciate natural environments (Chawla, 2007; Kalvaitis and Monhardt, 2015), preferring them to artificial environments (Simmons, 1994; Mahidin and Maulan, 2012; Berto et al., 2015). In spite of that, more than half of the world’s children have little chance of being outdoors and in contact with Nature (Clements, 2004), and most importantly, they can no longer play in Nature (Chawla, 2016). Children live in highly modified environments associated with low biodiversity (Turner et al., 2004); they have little independence and are not free to roam and explore (O’Brien et al., 2000). In urban environments, road traffic reduces children’s autonomy (Carver et al., 2008); coupled with parental concerns about neighborhood safety, these conditions further reduce the children’s desire and ability to play outdoors (Timperio et al., 2004). This being the case, children increasingly occupy their time with technological devices which tend to replace Nature as a playing and learning space (Pergams and Zaradic, 2006; Ballouard et al., 2011; Soga and Gaston, 2016).

Children losing their connection with Nature are not without consequences. It has a negative impact on their health and wellbeing (Samborski, 2010), leading to a higher risk of obesity (Wolch et al., 2011; Halonen et al., 2014), decreased ability for problem solving and risk assessment (Kuo and Taylor, 2004), and loss of motivation to protect Nature (Miller, 2005; Wells and Lekies, 2006). For their biophilia to be stimulated, children need frequent contact with Nature, initially with domestic Nature, and then extending the exploration to wild Nature (Hordyk et al., 2015). Children’s innate inclination to appreciate many forms of wild Nature can flourish only if it is adequately stimulated (Fattorini et al., 2017; Venturella and Barbiero, 2021). If biophilia is not stimulated in children, they tend to prefer domestic Nature (private gardens and courtyards), even when they have the freedom to access areas with high biodiversity (Hand et al., 2017a). This does not mean that today’s children are less biophilic, “but rather that their ability to act in this way has been curtailed” (Hand et al., 2017b). This appeared as evident in the experimental observations which led to the definition of the *Standard of Étroubles* (for more details, see Berto et al., 2015), where it emerged that, in a group of primary school children during a day spent in a wooded environment, the perception of restorativeness (presumably mediated by the fascination exerted by Nature) was increased, while the feeling of affiliation remained unchanged. A biophilic personality develops over time, and for the feeling of affiliation to grow, direct and frequent exposure to Nature is required (Venturella and Barbiero, 2021).

### Biophilic Design for a Biophilic Environment

Due to the Urban lifestyle, our contact with Nature has become less frequent (Turner et al., 2004). Nature continues to fascinate us, but our sense of affiliation with wild Nature has slackened (Miller, 2005). Our sporadic encounters with Nature are no longer enough to stimulate our biophilia, which tends to atrophy (Wilson, 1993; Barbiero, 2011). In the near foreseeable future, the phenomenon of disconnection from Nature will tend to accentuate. In 2007, urban population surpassed rural population for the first time in human history. Forecasts for 2050 are that 75% of the population will live in cities by then (Worldbank, 2019). From a certain point of view, this is good news. If human presence in rural environments decreases, it is foreseeable that wild Nature will tend to take over the spaces left behind. Larger habitats will increase the chances of survival of currently endangered wildlife species (Fahrig and Merriam, 1994; Fischer and Lindenmayer, 2007). However, Urban environments dwellers will have increasingly fewer opportunities to get in touch with Nature. Therefore, it becomes important to create Urban environments that will stimulate our biophilia as much as possible (Beatley, 2011; Hartig and Kahn, 2016; Söderlund, 2019). *Biophilic design* has been suggested as a way to meet this need (Kellert et al., 2008). The goal of biophilic design is to create artificial environments as similar as possible to natural ones, to ensure the positive effect that Nature has on people’s health and wellbeing (Söderlund, 2019; Browning and Ryan, 2020). Over the past 15 years, several biophilic design models have been suggested (see Table 2), which have often been implemented in advanced building certification systems (WELL, 2016a,b; LBC, 2017; LEED, 2018). Despite their specific differences, the criteria adopted by the various biophilic design models seem to respond to psychological needs matured over the course of evolution (for more details, see Bolten and Barbiero, 2020). In point of fact, the elements of biophilic design can be broken down into two fundamental groups following evolutionary adaptation principles developed by our species in the search for safe and resource-rich habitats (Orians, 1980, 1986). The first such group (light, protection and control, air, and views) seems to satisfy the theme of “searching for a safe place to live” (Buss, 2016); the element of this group is the basis of the savannah hypothesis (Orians, 1980, 1986). The second group of elements (greenery; curiosity; materials and finishing and colors) seems more linked to the theme “searching for resources and acquiring food” (Buss, 2016).

**Table 2:** Comparison of the most important features of biophilic design according to the most relevant studies.

Kellert, 2008	Browning et al., 2014	Kellert, 2018	Bolten and Barbiero, 2020
Natural light	Dynamic and diffuse light	Natural light	Light
Prospect and refuge	Prospect and refuge	Prospect and refuge	Protection and control
Air	Thermal and airflow variability	Air	Air
Views and vistas	Visual connection with nature	Views	Views
Plants	Visual connection with nature	Plants	Greenery
Curiosity and enticement	Mystery	–	Curiosity
Natural materials	Material connection with nature	Materials	Materials and finishing and colors

Source: Bolten and Barbiero (2020, modified), reprint with permission.

Although biophilic design research can rely on a robust evolutionary theoretical framework, it remains largely empirical (Kellert, 2018). There have been very few projects subjected to an experimental verification plan. Among them is the Biosphera Project of the Italian-Swiss company AktivHaus belonging to the Nexlogic Group. Biosphera Project is a research program unique for its kind because it is based on the creation of prototypes of transportable housing units. Being relatively mobile, the housing prototypes built so far – Biosphera 2.0 and Biosphera Equilibrium – have the advantage of being suitable for different environments: wilderness, rural, and burg. Starting in 2016, the Biosphera Project researchers have been collecting numerous experimental indications revealing the importance of biophilic design in promoting psychophysical wellbeing (Berto et al., 2020). These experimental results contributed to the creation of the Biophilic Quality Index (BQI; Berto and Barbiero, 2017b). The application of the BQI in a biophilic designed redevelopment project of the primary school in Gressoney-La-Trinité, Aosta Valley (Italy), introduced an experimental approach to biophilic design (Barbiero et al., 2017). That of Gressoney-La-Trinité is the first biophilic school where a three-year longitudinal study was conducted which highlighted the importance of restorative biophilic designed learning environments, that is, capable of supporting learning processes and reinforcing the bond of affiliation with Nature (Venturella and Barbiero, 2021). Continuous and long-lasting contact with Nature allows children to establish a deeper affiliation with Nature and lays the foundations for pro-environmental behavior in adults (Berto and Barbiero, 2017a).

## Conclusion

The aim of this review was to explain our response to Nature against the background of our evolutionary past. Natural selection favors traits which bring advantages in struggle for survival. Biophilia consists of learning rules that facilitate effective contact with Nature; this is its main evolutionary advantage. The fascination exerted by Nature and affiliation with Nature are the biophilic constructs identified by Wilson (2002), which, being able to be operationalized (Barbiero and Berto, 2018), allow us to relate their evolutionary roots with the positive effects that Nature exerts both on a physiological and cognitive level. The evolutionary explanation can also account for the different experiences of Nature depending on environmental typology (wilderness, rural, and burg), which in turn reflects the phylogenetic typology of human experience with Nature (Paleolithic, Neolithic, and Urban).

Although there is little doubt that biophilia has an evolutionary origin, some researchers disagree that the attraction that humans feel for Nature has become fixed over the course of its evolution and criticize the evolutionary interpretation of biophilia (Joye and De Block, 2011; Joye and Van den Berg, 2011; Haga et al., 2016). To this approach, individual’s response to natural stimuli does not depend on the stimulus characteristics, whereas on the meaning that individuals assign to it, accordingly, individual’s positive response to Nature (including preference and perceived restoration) has been learned and depends on positive emotional associations. These observations are not in contrast with the biophilic hypothesis proposed here. The hypothesis that behavior depends on both biological/hereditary and environmental/cultural factors and that these are not additive but interactive is not new; what is new is the role recognized to cognitive and affective processes as interacting variables capable of significantly acting on biological and environmental factors (Caprara and Gennaro, 1994). Biophilia consists of weak learning rules, which require contact and experience before affiliation with Nature is consolidated. It is therefore foreseeable that, as one learns from experiencing Nature, the feeling of affiliation with Nature will deepen, triggering a virtuous process involving concern for the environment and pro-environmental behavior (Barbiero and Berto, 2018). For this reason, it can be assumed that biophilic design can generate those positive emotions associated with Nature, reproducing and incorporating in the design of the built environment the direct and indirect experiences of Nature (Kellert, 2018), which derive from evolutionary stratification of biophilic experiences.

Biophilia is not a cultural by-product but a “useful trait,” that is, a characteristic that directly contributed to self-preservation and reproduction, which provided us useful information about the natural environment, seeking out and exploring the novel and extraordinary environment we had to face. Biophilia steers our relationship with Nature, including preference for natural environments that can aid recovery from attentional fatigue and psycho-physiological stress (Berto, 2014; Berto et al., 2018). In brief, from the evolutionary point of view, biophilia is a shared predisposition to recognize that a certain habitat reflects the adaptations designed by natural selection aimed to help us to choose the place where to live.

## Author Contributions

All authors listed have made a substantial, direct and intellectual contribution to the work, and approved it for publication.

### Conflict of Interest

The authors declare that the research was conducted in the absence of any commercial or financial relationships that could be construed as a potential conflict of interest.
